# LncRNA PVT1 influences breast cancer cells glycolysis through sponging miR-145-5p

**DOI:** 10.1007/s13258-023-01368-8

**Published:** 2023-03-20

**Authors:** Huan Qu, Xingxing Li, Fei Chen, Min Zhang, Xun Lu, Yun Gu, Mingming Lv, Cheng Lu

**Affiliations:** 1 Department of Breast, Women’s Hospital of Nanjing Medical University, Nanjing Maternity and Child Health Care Hospital, 123rd Tianfei Street, Mochou Road, Nanjing, 210004 China; 2 Department of Health Management Centre, School of Medicine, Zhongda Hospital, Southeast University, Nanjing, 210009 China; 3 School of Public Health, Yale University, New Haven, CT 06520 USA; 4 Department of Pathology, Nanjing Maternity and Child Health Care Hospital, Women’s Hospital of Nanjing Medical University, Nanjing, 210004 China

**Keywords:** Breast cancer, LncRNA PVT1, miR-145-5p, Glycolysis

## Abstract

**Background:**

Long-non-coding RNA PVT1 (lncRNA PVT1) can be used as an oncogenic regulatory non-coding RNA (ncRNA) for many cancers. However, its function and mechanism in breast cancer (BRCA) are still not clearly elucidated.

**Objective:**

We attempt to explain the mechanism of PVT1’s role in breast cancer from different perspectives.

**Methods:**

We analyzed the expression of PVT1 and its correlation with the breast cancer related clinical data in the The Cancer Genome Atlas (TCGA) database. We used PVT1 overexpression and knockdown lentivirus to infect breast cancer MDA-MB-231 cell line for cell function verification, in vitro using CCK-8 to measure proliferation, flow cytometry to measure apoptosis, transwell test to measure invasion and migration ability, detecting cell extracellular acidification rate (ECAR) to assess glycolysis metabolism and explore the biological functions of PVT1 in breast cancer cells. Transcriptome sequencing was used to analyze the changes of related genes in cells after overexpression of PVT1. In vivo we used a xenograft model to study the effect of PVT1 on breast cancer.

**Results:**

PVT1 was up-regulated in breast cancer tissues and was positively correlated with the clinical stage of breast cancer patients. Overexpression of PVT1 in vitro promoted cell proliferation, migration and invasion, and promoted tumor growth in vivo. Knockdown of PVT1 led to the opposite biological consequence. Further bioinformatics analysis showed that PVT1 changes the glycolysis metabolism of tumors through regulation of glycolysis-related genes. In addition, the expression of miR-145-5p is negatively correlated with PVT1. We consider the possibility of PVT1 promoting cell proliferation and metastasis by regulating the aerobic glucose metabolism in breast cancer cells through sponging the miR-145-5p.

**Conclusion:**

Our results reveal a potential pathway for competing endogenous RNA to regulate breast cancer glucose metabolism. PVT1 regulates glycolysis related genes expression by competitively binding to endogenous miR-145-5p in breast cancer cells to change the metabolic phenotype. This may Provide new ideas for precise molecular therapy targets for breast cancer.

## Introduction

Breast cancer has always been a common malignant tumor for women worldwide. The latest cancer statistics showed that breast cancer accounts the most number of new onset cancers in women (Siegel et al. 2021). The high incidence of breast cancer not only severely damages women’s health and lives, but also imposes an immeasurable economic burden on the family and society (Sung et al. 2021). It is an urgent public health problem facing society today. Due to chemotherapy resistance and endocrine therapy resistance, the overall survival rate of breast cancer patients still needs to be improved (Derakhshani et al. 2020; Emens 2018; Hanker et al. 2020). Therefore, finding new therapeutic targets and the mechanism is still the theme issue that needs to be solved urgently.

It was estimated that at least 75% of human genome transcripts can be transcribed into non-coding RNAs (Djebali et al. 2012). Long non-coding RNA (lncRNA) is a subtype of non-coding RNA (ncRNA) transcripts longer than 200 nucleotides, involved in a series of biological and pathological processes, including angiogenesis, osteoarthritis, diabetes, tumor occurrence and development (Huarte 2015; Peng et al. 2017; Qian et al. 2019; Zhao et al. 2020). Long-non-coding RNA PVT1 has the effect of promoting tumorigenesis and development in a variety of malignant tumors, such as gastrointestinal cancer (Martínez-Barriocanal et al. 2020; Zhao et al. 2018), cholangiocarcinoma (Chen et al. 2019). Moreover, the functional diversity of PVT1 in tumors has been gradually proved, which not only directly regulates the malignant biological behaviors such as proliferation, invasion and migration, but also plays an indirect role in tumor microenvironment, metabolism and other aspects. Therefore, we attempt to explain the mechanism of PVT1’s role in breast cancer from different perspectives.

We first identified that changing the expression level of PVT1 in breast cancer cells can change the level of glycolysis of the tumor cells, thereby promoting the biological behavior of the tumor. Overexpression of PVT1 in vitro promotes cell proliferation, migration and invasion, and promotes tumor growth in vivo. Knockdown of PVT1 has the opposite biological function. Further bioinformatics analysis showed that PVT1 changes the glycometabolism of tumors through its regulation of glycolysis-related genes. We consider PVT1 promoting cell proliferation and metastasis by regulating the aerobic glucose metabolism in breast cancer cells through sponging miR-145-5p.

## Materials and methods

### Cell culture and stable cell lines construction

MDA-MB-231 and HEK-293T cell lines were purchased from Shanghai Cell Bank of the Chinese Academy of Sciences (Shanghai, China). Both cell lines were grown in Dulbecco’s Modified Eagle’s Medium (Gibco, USA) supplemented with 10% FBS and 1% penicillin-streptomycin mixture and incubated at 37 °C with 5% CO_2_. To construct PVT1 stably overexpressed and knockdown breast cancer cells, pLVX-PVT1-EF1α-IRES-EGFP-PGK-puro and pLVX-shPVT1-zsGREEN-PGK-puro was used as vector plasmid. GM easy^™^ Lentiviral Packaging Kit was used to transfected into HEK293T cells along with the vector plasmid. Then change the medium after 18 h and collect the virus-containing supernatant after 48 h. The collected virus supernatant was used to detect titer and infect MDA-MB-231 cells. Then the cells were treated with puromycin (2 ug/ml) to select PVT1 stably overexpressed or knockdown cells. The express efficiencies of PVT1 in MDA-MB-231 cells were measured by qRT-PCR. The sense sequences of PVT1 sh-RNA were 5′-AGTGTCCTGGCAGTAA-3′ and 5′-ATCGTAATGGGTTGAA-3′.

### Cell proliferation assay

We used the CCK8 assay and EdU(5-ethynyl-2′-deoxyuridine) assay to assess cell proliferation. For CCK8 assay, MDA-MB-231 cells were cultured in 96-well plates at 3000 cells/well. After 0 h, 24 h, 48 and 72 h, the final ratio of adding CCK8 reagent to each well is 10% (v/v). 2 h later, the absorbance of each well at 450 nm was measured by a microplate reader (H4, Synergy).

### Transwell assay

Cellular invasion ability of indicated breast cancer cells was evaluated using transwell assay with Matrigel (Sigma, St Louis MO, USA). Indicated MDA-MB-231 cells stably overexpressed negative control (NC), PVT1 cells were resuspended in serum-free medium and plated in Transwell upper chambers (Corning Inc, New York, USA). DMEM medium with 10% FBS was plated to the lower chambers. After further incubation for 48 h, the cells that invaded into the bottom side of the chambers were fixed and stained with 0.1% crystal violet. The results were collected using a microscope. Four fields of view were randomly selected to calculate the relative invasion.

### Cell apoptosis analysis of cells

MDA-MB-231 cells were harvested with EDTA-free trypsin for apoptosis analysis. The cells were resuspended in 500 µl of the binding buffer and stained with 10 µl of Annexin V/PI (BD Bioscience, USA) at room temperature for 10 min. All samples were analyzed using flow cytometry by Kaluza software (Beckman Coulter). The data acquisition (20,000 events collected per sample) was performed following the manufacturer’s instructions.

### RNA-seq analysis

Total RNA was extracted from OENC and PVT1 overexpressed MDA-MB-231 cells by using TRIzol reagent (Thermo Fisher Scientific). The concentration of RNA was determined by measuring the absorbance ratio at 260/280 nm by using spectrophotometer (NanoDrop ND-1000 Thermo Scientific). The purified total RNA was sent to BGI (Shenzhen China) and used to construct the mRNA library. The RNA-seq data was analysed by the BGI bioinformatics platform. The second-generation transcriptome sequencing results was uploaded on the NCBI Gene Expression Omnibus (GEO) database with the accession number GSE67994. The sequences of PVT1 primer for qRT-PCR were TGGAATGTAAGACCCCGACTCT (forward) and GATGGCTGTATGTGCCAAGGT (reverse). The miR-145-5p mimics sequences were as follows: sense: 5′-GUCCAGUUUUCCCAGGAAUCCCU-3′. The miR-145-5p sequences for qRT-PCR was 5′-CCGGTCCAGTTTTCCCAGGAATCCCT-3′ (forward).

### Glycolysis stress test

Seahorse XF24 Glycolysis Analyzer (Seahorse Bioscience, MA, USA) was used to analysis extracellular acidification rate (ECAR) to evaluate the effect of PVT1 depletion on glycolysis stress. For ECAR analysis, glucose, oligomycin, and 2-deoxyglucose were sequentially added in special medium. Glucose was first injected into the medium and catabolized to lactate and ATP with a corresponding increased ECAR value. Then, oligomycin was injected, which inhibited mitochondrial ATP production and shifted the energy production to glycolysis, with the corresponding increase in ECAR. The ECAR was reported in milli-pH (mpH) units per minute.

### Murine xenograft assay

All total of 36 4-week-old BALB/c nude mice (Charles River Laboratories, Beijing, China) were subcutaneously inoculated into the back with 1 × 10^7^ MDA-MB-231 PVT1 stably overexpressed or knockdown cell. 36 BALB/c nude mice were randomly assigned to 6 groups (6 per group) for the injection. The subcutaneous xenografts were resected and weighed. Then, the subcutaneous tumors were fixed with formalin and made into paraffin-embedded sections for the routine immunohistochemistry (IHC) assay. The murine xenograft assay was approved by the Institutional Animal Care and Use Committee of Nanjing Medical University (Nanjing, China)(Approval Number: IACUC-2012016).

### Immunohistochemistry

The tumors were stained with Ki67 and MMP9 to measure their levels. After blocking, the tumors sections were incubated with primary antibodies (anti-KI67, CST #2586; and anti-MMP9, CST #13,667) overnight, followed by incubation with secondary antibodies and further treated with diaminobenzidine and counterstained with hematoxylin. All tissues were observed and photographed with a microscope (Carl Zeiss, Oberkochen, Germany).

### Statistical analysis

The quantitative data are expressed as the means ± SD. The values were compared using Student’s *t *test or one-way ANOVA. All of the statistical analyses were performed using SPSS 13.0 software (Chicago, IL, USA). A p-value of < 0.05 was considered statistically significant.

## Results

### PVT1 was highly expressed in breast cancer

**Fig. 1:**
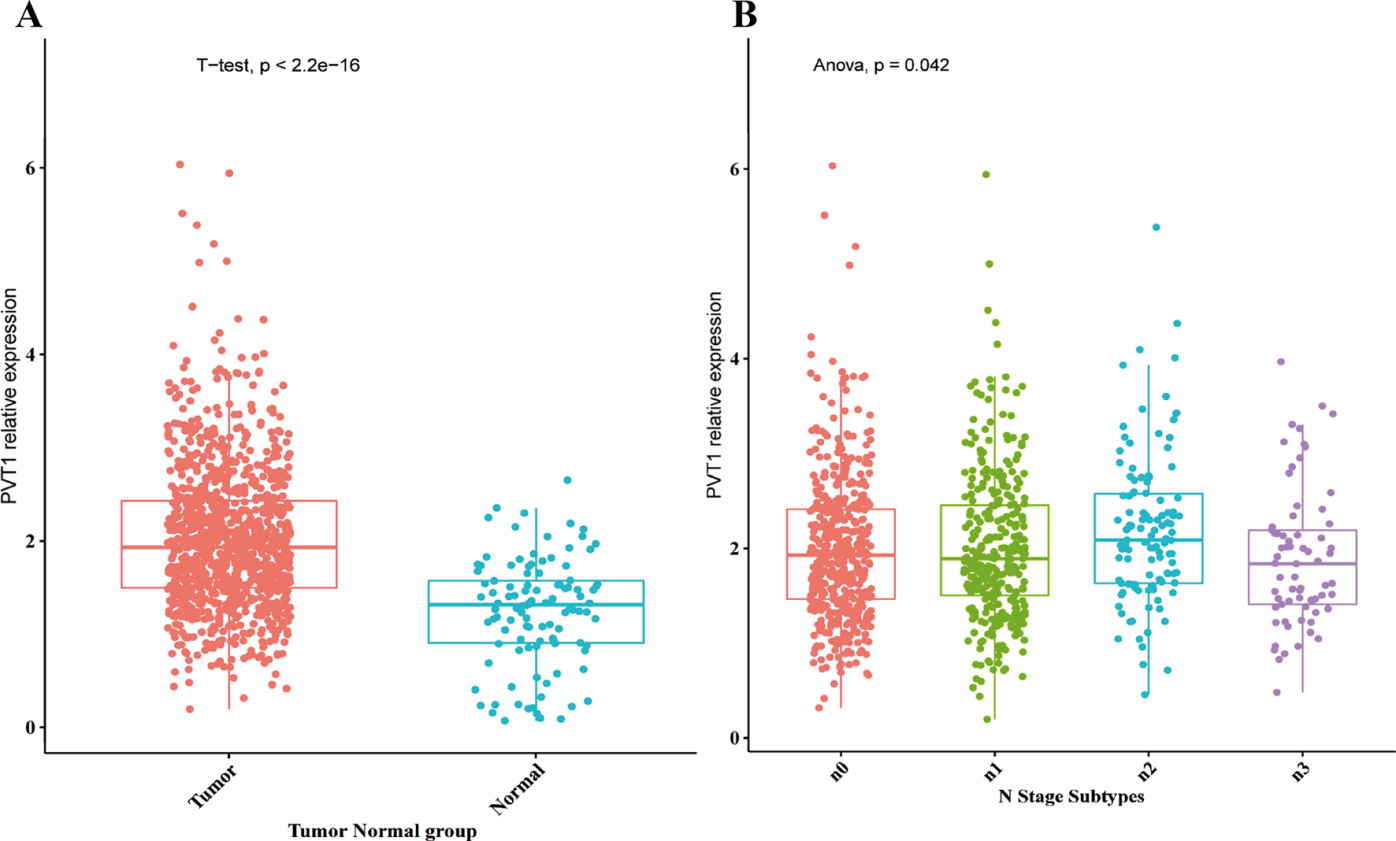
Analysis of breast cancer related clinical data from TGCA database. **A** PVT1 is highly expressed in breast cancer tissues (P < 0.05). **B** PVT1 is closely related to the lymph node metastasis of breast cancer patients (P < 0.05)

We first investigated the expression of PVT1 in TCGA database which included 1085 breast invasive carcinoma (BRCA) tissues and 170 normal tissues (Berger et al. 2018). As shown in Fig. 1A, the expression intensity of PVT1 was significantly increased in BRCA tissues than normal tissues (P < 0.01). Moreover, the expression level of PVT1 is closely related to the lymph node metastasis of breast cancer patients (P < 0.05, Fig. 1B).

### Overexpression of PVT1 promoted cell proliferation, invasion

**Fig. 2:**
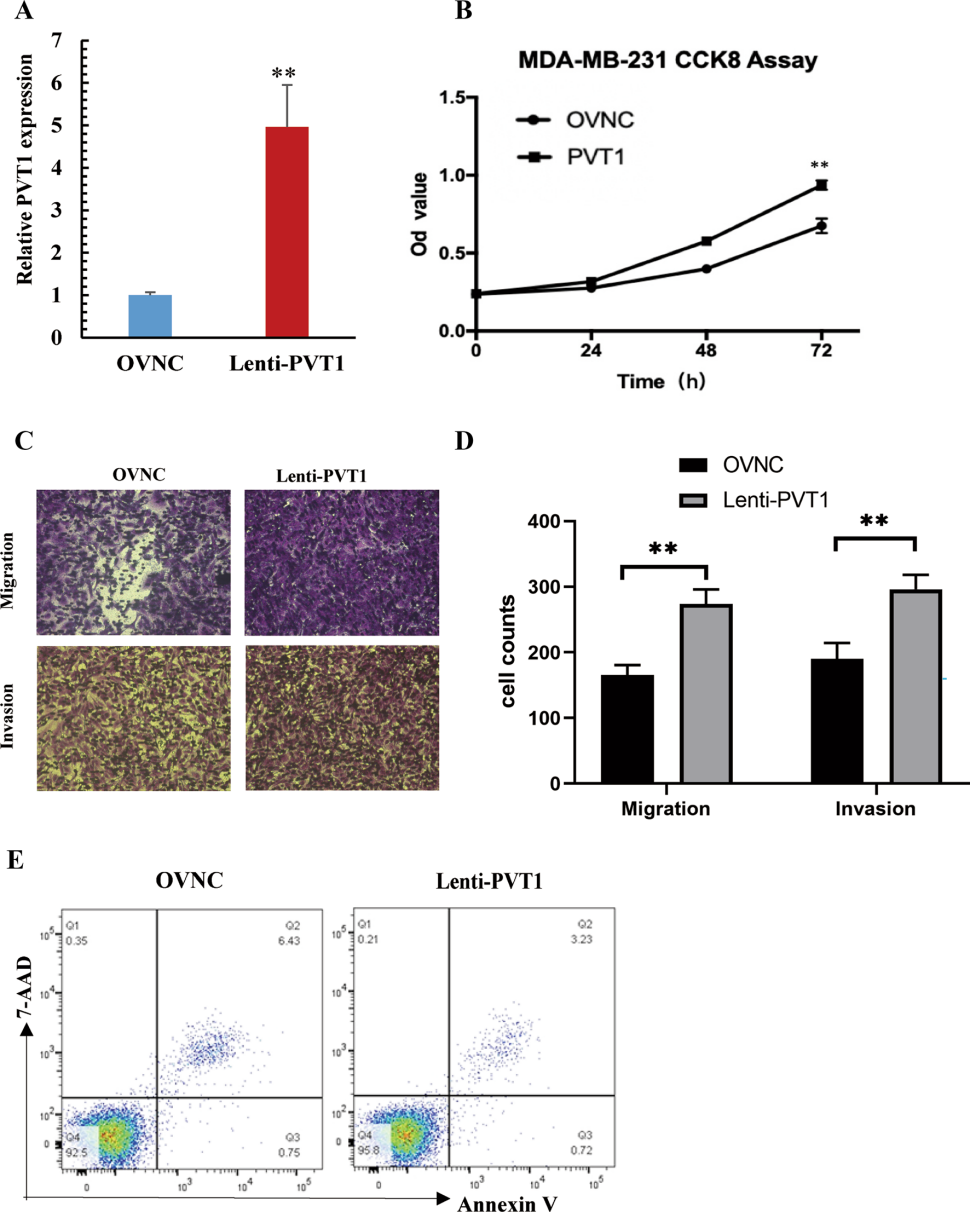
Overexpression of PVT1 promotes tumor proliferation, enhances invasion and migration of breast cancer cell lines. **A** PVT1v overexpression efficiency. **B** CCK-8 assay showed PVT1 promoted cell proliferation in MDA-MB-231 cells. **C**, **D** Overexpression of PVT1 significantly increased migration and invasion ability of MDA-MB-231 cells. **E** Flow cytometry experiment showed that apoptotic cell number was similar between PVT1 overexpressed cells and normal control in MDA-MB-231 cells. *P < 0.05, **P < 0.01, ***P < 0.001

To assess the function of PVT1 in BRCA, we established a PVT1 stable overexpression cell line using lentivirus infection in MDA-MB-231 cells and tested the overexpression efficiency (Fig. 2A). CCK-8 assay was undertaken to measure cell proliferation ability. It was observed that PVT1 promoted cell proliferation in MDA-MB-231 cells (Fig. 2B). Transwell migration and invasion assay was applied to evaluate cell migration and invasion. Overexpression of PVT1 significantly increased migration and invasion ability of MDA-MB-231 cells (Fig. 2C, D). We applied Annexin V-7AAD staining and flow cytometric analyses to detect cell apoptosis. The results showed that the ratio of Annexin V^+^ 7-AAD^−^ apoptotic cell were similar between PVT1 overexpressed cells and normal control (Fig. 2E). Thus, these findings suggested that overexpression of PVT1 promoted cell proliferation and invasion of breast cancer cells.

### Knockdown of PVT1 inhibited cell proliferation, invasion

**Fig. 3:**
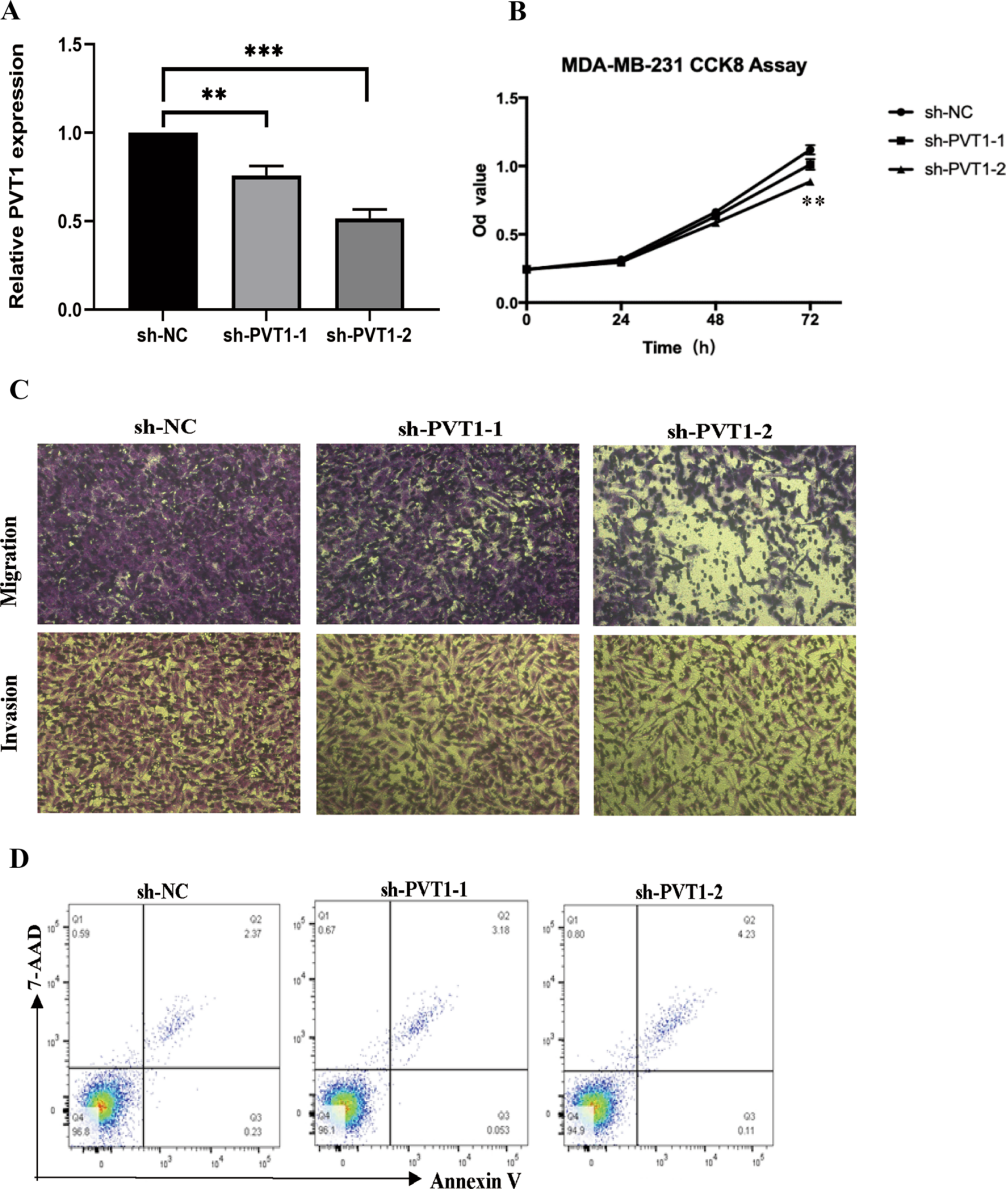
Knockdown of PVT1 level inhibits tumor proliferation of breast cancer cell lines, enhances invasion and migration. **A** Knockdown efficiency. **B** CCK-8 assay was undertaken to measure cell proliferation ability. **C** Knockdown of PVT1 expression level significantly decreased migration and invasion ability of MDA-MB-231 cells. **D** Flow cytometry experiment showed that apoptotic cell number has no significant difference within NC and two sh-PVT1 groups in MDA-MB-231 cells. *P < 0.05, **P < 0.01, ***P < 0.001

To further assess the function of PVT1 in breast cancer, we established a MDA-MB-231 cell line with stably PVT1 gene silencing by lentivirus-mediated short hairpin RNA interference and tested the knockdown efficiency. The cells transfected with sh-PVT1-2 obtained more knockdown efficiency (Fig. 3A). CCK-8 assay was undertaken to measure cell proliferation ability. It was observed that knockdown of PVT1 expression level inhibited cell proliferation in MDA-MB-231 cells (Fig. 3B), and decreased migration and invasion ability of MDA-MB-231 cells (Fig. 3C). The apoptosis results showed that Annexin V^+^7AAD^−^ apoptotic cell number has no significant difference within NC and two sh-PVT1 groups (Fig. 3D). Thus, these findings suggested that knockdown of PVT1 inhibited cell proliferation and invasion of breast cancer cells.

### PVT1 could promote breast cancer progression via enhance glycolysis by sponging miR-145-5p

**Fig. 4:**
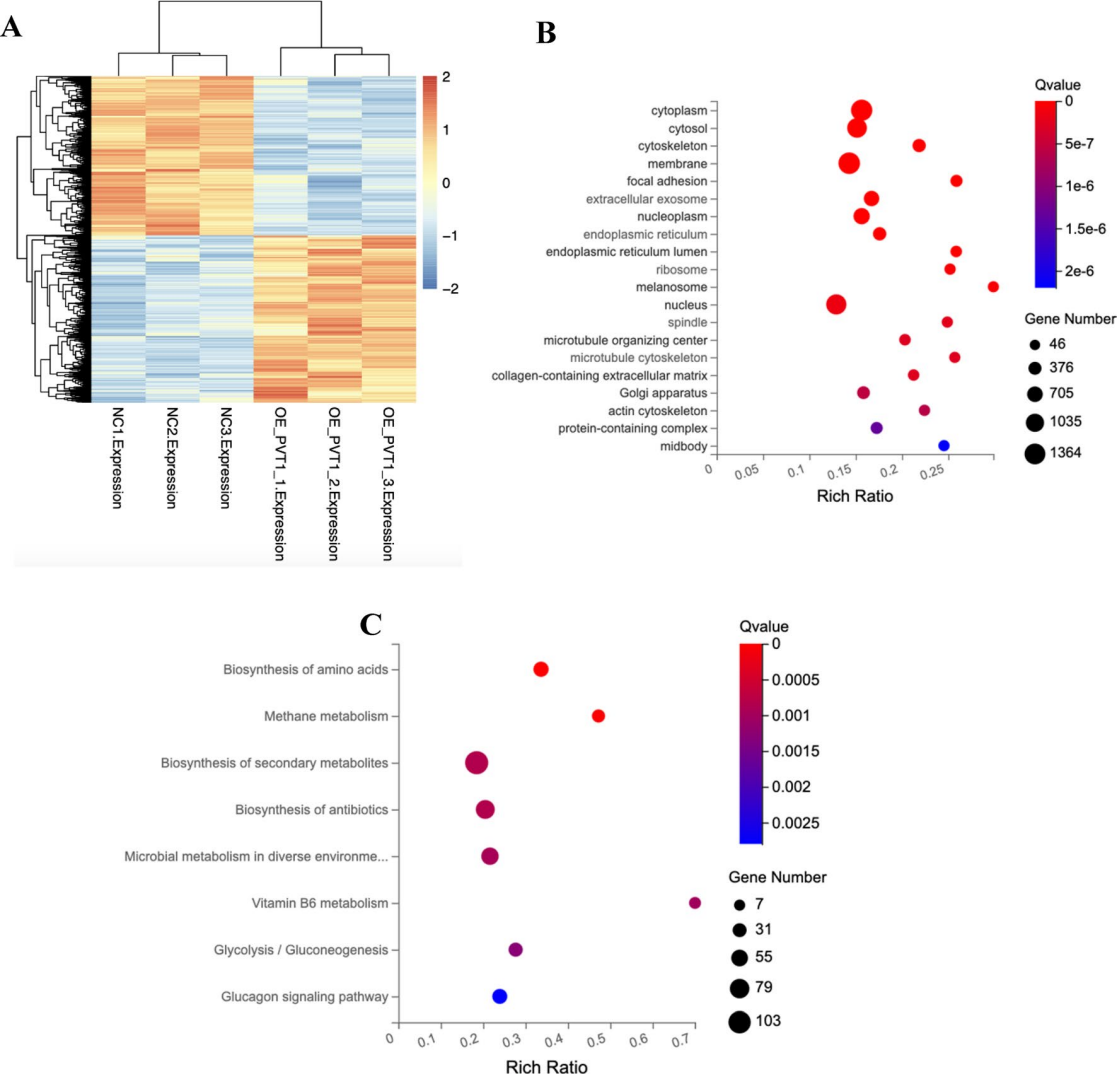
Clustering and enrichment analysis of second-generation transcriptome sequencing results of PVT1 overexpression stable transgenic strain and control group. **A** RNA-Seq analysis showed the different expressed gene between overexpression NC and PVT1 overexpression group. **B** GO analysis of the differential expressed genes most involved in cytoplasm. **C** KEGG pathway enrichment analysis of the differential expressed genes showed PVT1 may involve in the metabolism of many substances, including glycolysis

**Fig. 5:**
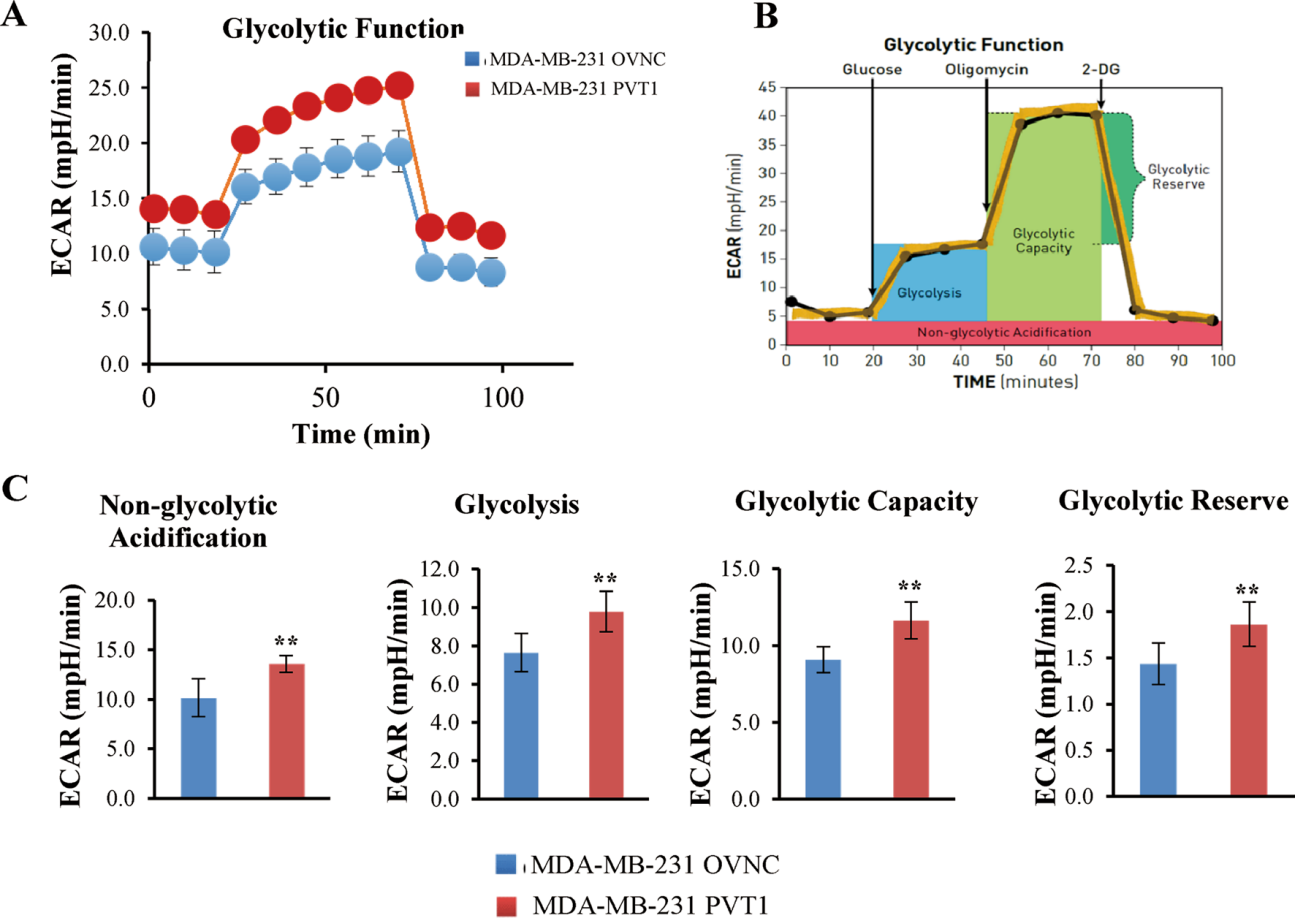
PVT1 overexpression group can significantly enhance the glycolytic ability of breast cancer cells (**A**). Extracellular Acidification (ECAR) profile of glycolysis stress test on OVNC and PVT1 overexpression group. **B** Illustration of glycolysis stress test mode. **C** PVT1 overexpression group significantly enhance the glycolytic ability of non-glycolytic Acidification, glycolysis, glycolytic capacity and glycolytic reserve

**Fig. 6:**
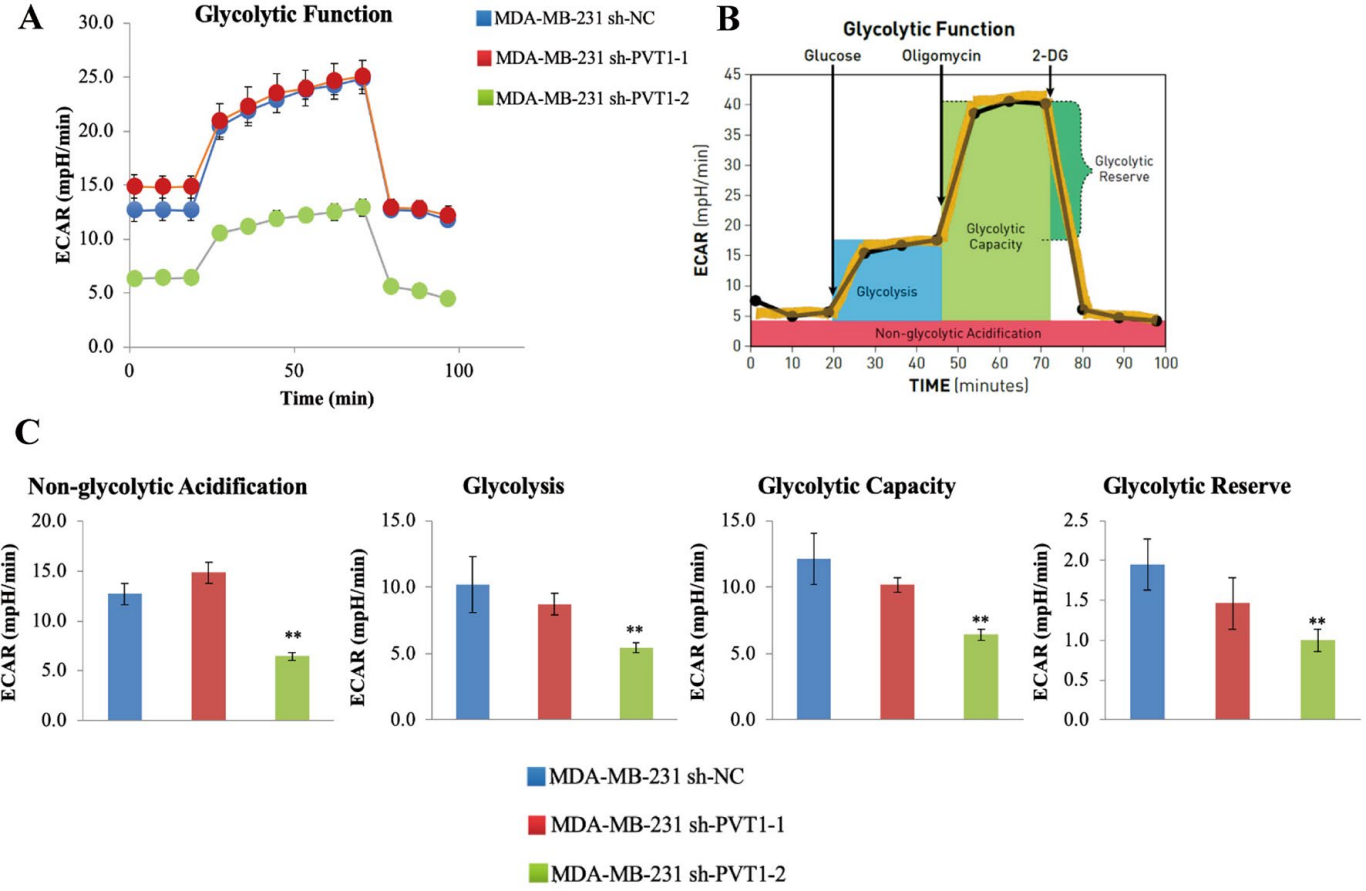
PVT1 knockdown group can significantly inhibit the glycolytic ability of breast cancer cells. **A** Extracellular Acidification (ECAR) profile of glycolysis stress test on shNC and PVT1 knockdown group. **B** Illustration of glycolysis stress test mode. **C** sh-PVT1-2 knockdown group significantly inhibit the glycolytic ability of non-glycolytic Acidification, glycolysis, glycolytic capacity and glycolytic reserve

**Fig. 7:**
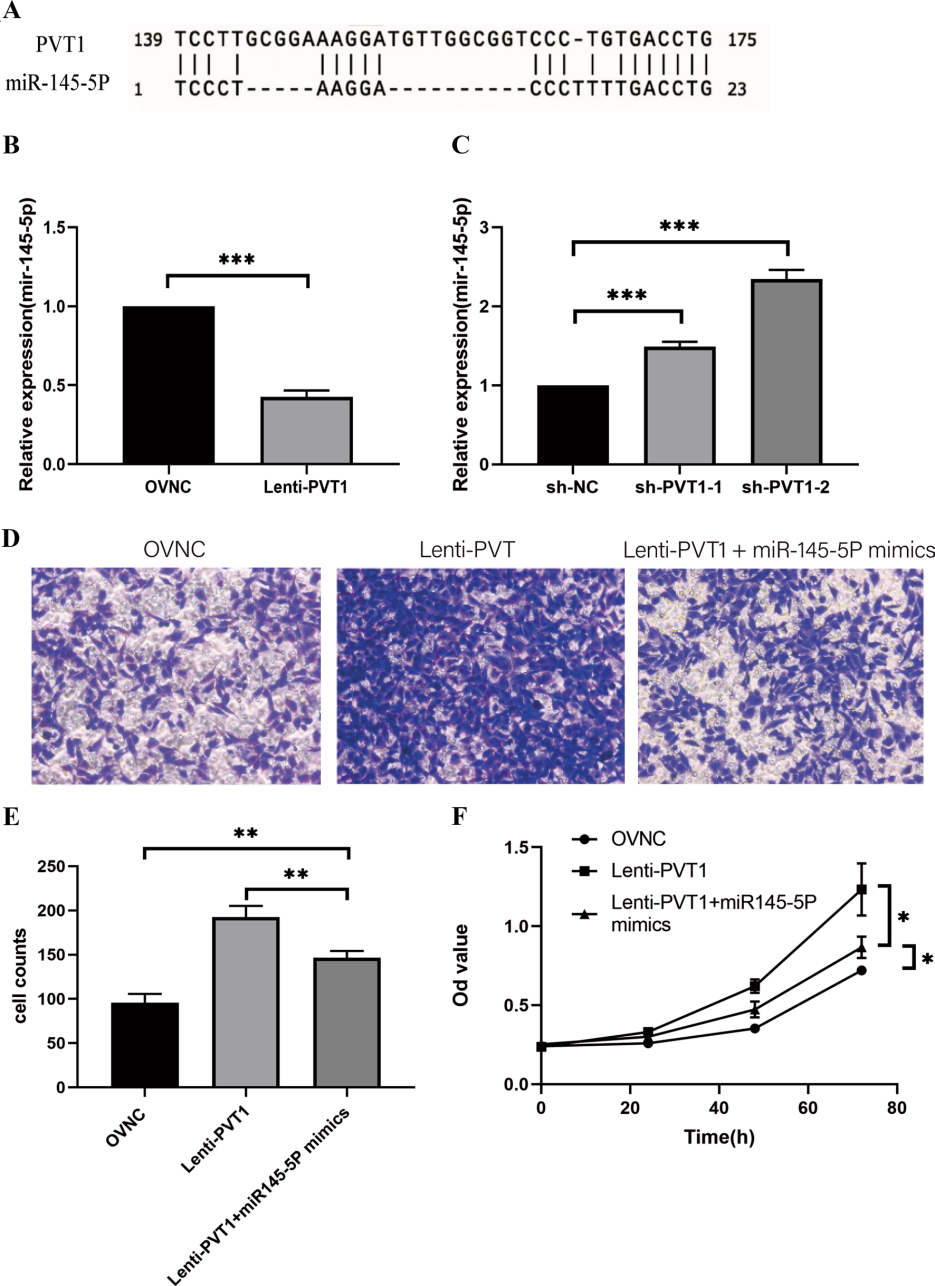
The interaction between PVT1 and miR-145-5p. **A** Predicted binding sites of miR-145-5p and PVT1. **B** MiR-145-5p has the significant downregulation after PVT1 overexpression. **C** MiR-145-5p has the significant upregulation after the silence of PVT1. **D**, **E** PVT1 overexpressed breast cancer cells showed decreased migration ability after being transfected with miR-145-5p mimics. **F** Transfecting with miR-145-5p mimics in PVT1 overexpressed breast cancer cells inhabited the cell proliferation. *P < 0.05, **P < 0.01, ***P < 0.001

To elucidate the molecular mechanisms underlying the effects of PVT1 on breast cancer cells, we performed RNA-seq and examined the mRNA expression profiles after overexpressing PVT1 in breast cancer cell line MDA-MB-231. Through cluster analysis (heatmap), GO and KEGG enrichment analysis (Fig. 4A, C), and these results indicated that most of the DEGs are localized in the cytoplasm and function in multiple metabolism pathways. Among them, the glycolysis pathway gene enrichment changes significantly (Rich ratio: 0.277, P value: 0.00005622753, Q value: 0.001275662). Regarding the enrichment results of glycolysis-related pathways, we used the seahorse detection protocol to detect the correlation between PVT1 levels in breast cancer cells and glycolytic stress and found that the PVT1 overexpression group can significantly enhance the glycolysis ability of breast cancer cells (Fig. 5A–C), while the glycolytic ability of the PVT1 knockdown group was significantly reduced (Fig. 6A–C). We used the Starbase database (http://starbase.sysu.edu.cn/index.php) to predict the miRNA that has binding sites with PVT1 (Fig. 7A) and selected 4 of them to perform qPCR to verify the changes in expression. We found that miR-145-5p had the significant downregulation after PVT1 overexpression (Fig. 7B), and miR-145-5p was significantly upregulated after silencing PVT1 (Fig. 7C). To further prove that PVT1 affects the cell proliferation and migration through sponging mir-145-5p, we transfected PVT1 overexpressed cells with miR-145-5p mimics. After transfection, the migration and proliferation were significantly decreased (Fig. 7D–F).

### PVT1 promoted breast cancer growth in vivo

**Fig. 8:**
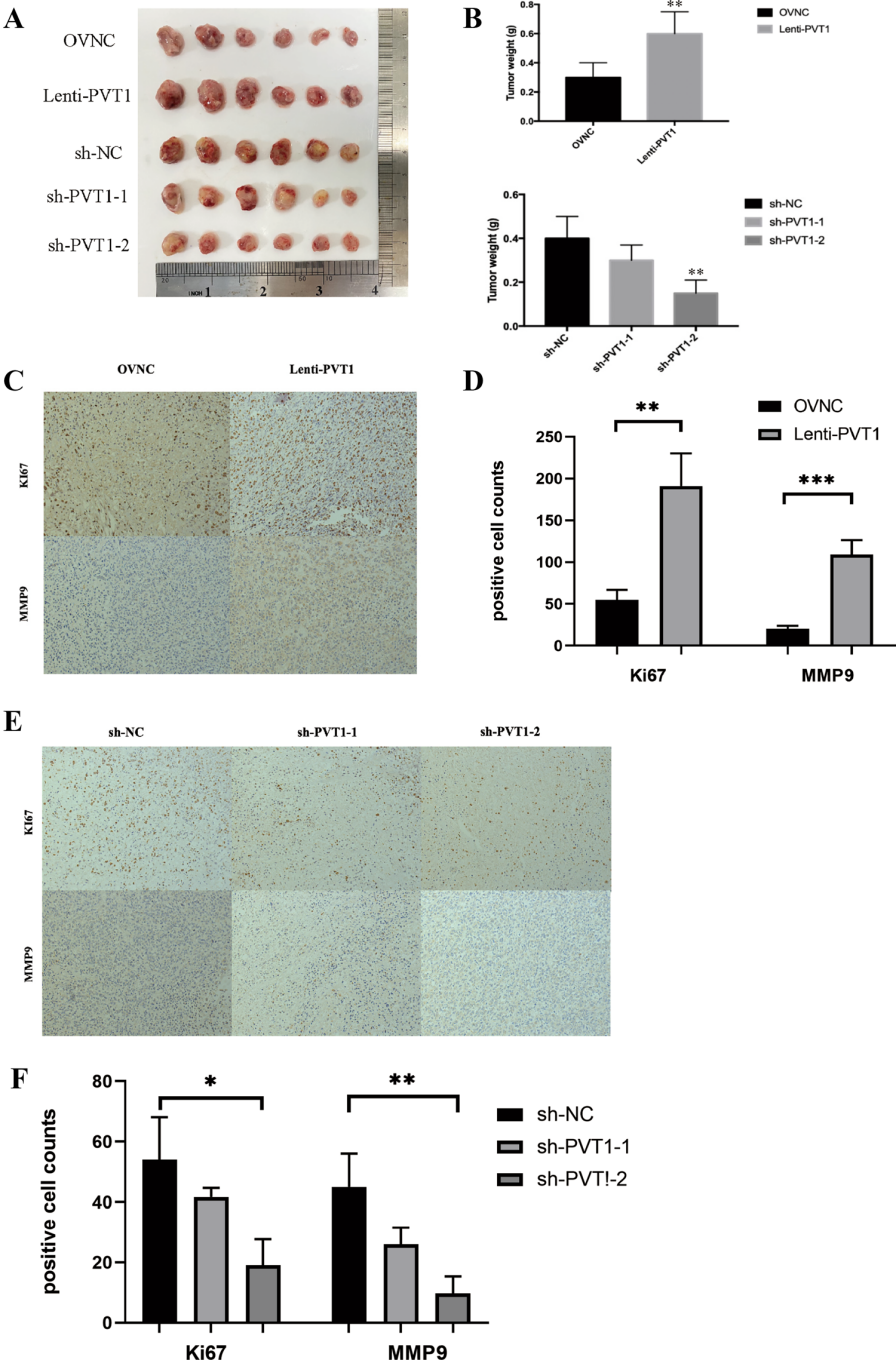
PVT1 tumor formation experiment in nude mice in vitro. **A** The overexpression of PVT1 promoted the formation of tumors in vitro. **B** The tumor size and quality were significantly higher than those of the control group. **C**–**F** Immunohistochemical results showed that KI67 and MMP9 had a higher positive rate in the PVT1 overexpression cell line and had a lower positive rate in PVT1 knockdown cell line. *P < 0.05, **P < 0.01, ***P < 0.001

To elucidate the function of PVT1 in breast cancer in vivo, PVT1 stably overexpressed, knockdown-expressed and control MDA-MB-231 cells were subcutaneously inoculated into nude mice. The subcutaneous xenograft growth was significantly increased by overexpressing PVT1 (Fig. 8A, B). The cell line with stable knockdown of PVT1 has significantly lower tumor diameter and weight than the control group (Fig. 8A, B). Immunohistochemical results showed that KI67, a proliferative antigen marker, and MMP9, an cell invansion maker, had a higher positive rate in the PVT1 overexpression cell line (Fig. 8C, D) and had a lower positive rate in PVT1 knockdown cell line (Fig. 8E, F). The results of immunohistochemistry were consistent with those of assays in vitro. Taken together, these findings showed that PVT1 promoted breast cancer cell proliferation and growth in vivo.

## Discussion

Various lncRNAs have been shown to be promising targets for tumor treatment in recent studies (Bhan et al. 2017). The expression of oncogenic lncRNA can be controlled by targeted small molecule inhibitors, RNAi technology, etc. to control tumor progression. At the same time, enhancing the expression of tumor suppressor lncRNAs can also be used as a gene therapy for cancer (Wang et al. 2019). Therefore, more researches are needed to explicate the function and mechanism of lncRNAs (Bhan et al. 2017).

Recently, a large number of studies have shown that lncRNA PVT1 is closely related to the occurrence and development of many malignant tumors. PVT1 has been reported to have obvious cancer-promoting effects in gastric cancer (Zhao et al. 2018), colorectal cancer (He et al. 2019), pancreatic cancer (Zhou et al. 2020a) and breast cancer (Tang et al. 2018). By inhibiting miR-143, it can promote gallbladder cancer (Chen et al. 2019). However, there are few studies on the relationship between PVT1 and tumor cells metabolism. Our study found that PVT1 is highly expressed in breast cancer and is closely related to the patient’s lymph node metastasis. Further research found that PVT1 can promote the proliferation, invasion and migration of triple-negative breast cancer cells.

It is recognized that tumor cells need to adjust their metabolism to adapt to the environment of nutritional deficiency (Yang et al. 2020). At present, some research believe that the metabolic mode of tumor is the Achilles heel of tumor (Ganapathy-Kanniappan and Geschwind 2013). Changing the metabolism of tumors through drugs or biological methods can exert the function of tumor inhibition(Lin 2020).The Warburg effect is a unique metabolic change in tumor cells. The ability of tumor cells to anaerobic glycolysis is enhanced, and there is still a strong glycolysis level even under aerobic conditions (Ganapathy-Kanniappan and Geschwind 2013). This feature can enhance tumor chemo-resistance, invasion and migration, and maintenance of tumor microenvironment (Orang et al. 2019). Our RNA-seq analysis showed that PVT1 may promote tumor development by changing metabolism of cells substance, especially glycolysis. Our metabolism experimental data also showed that overexpression of PVT1 can enhance breast cancer cells glycolysis. Indicating that PVT1 can be used as a target for breast cancer metabolism targeted therapy. In the results, PVT1 was silenced PVT1 by using two different shRNA. The expression levels were decreased approximately 30% for sh-PVT1-1 and 50% for sh-PVT1-2. This may explain that only cells silenced by sh-PVT1-2 had significant changes in glycolysis stress experiment. And cells silenced by sh-PVT1-2 has more significant difference in proliferation and migration than cells silenced by sh-PVT1-1.

MiR-145-5p is a kind of micro-RNA that plays a tumor suppressor function in a variety of tumors, and is reported to play a vital role in inhibition of breast cancer (Bellissimo et al. 2019), gastric cancer (Zhang et al. 2020; Zhou et al. 2020b) and colorectal cancer (Niu et al. 2019). The sponging effect of lncRNA is a common mechanism for long non-coding RNA to function. It can sponge the corresponding miRNA and cause the loss of miRNA function. Our research results show that overexpression of PVT1 can significantly reduce the expression of mir-145-5p, considering that there may be sponging adsorption, this data has also been verified in the database and related literature.

In summary, our results reveal a potential pathway for competing endogenous RNA to regulate breast cancer glucose metabolism. PVT1 regulates glycolysis related genes expression by competitively binding to endogenous miR-145-5p in breast cancer cells to change the metabolic phenotype. This may provide new ideas for precise molecular therapy targets for breast cancer.
